# Indirect Temperature Measurement in High Frequency Heating Systems

**DOI:** 10.3390/s21072561

**Published:** 2021-04-06

**Authors:** Alexander Oskolkov, Igor Bezukladnikov, Dmitriy Trushnikov

**Affiliations:** 1 Department of Welding Production, Metrology and Technology of Material, Perm National Research Polytechnic University, 29 Komsomolsky Prospect, 614990 Perm, Russia; trdimitr@yandex.ru; 2 Department of Automation and Telemechanics, Perm National Research Polytechnic University, 29 Komsomolsky Prospect, 614990 Perm, Russia; corrector@at.pstu.ru

**Keywords:** FFF, FDM, 3D-printing, induction heating, HF heating, indirect measurement, temperature measurement, eddy-current, resonance, regression analysis

## Abstract

One of the biggest challenges of fused deposition modeling (FDM)/fused filament fabrication (FFF) 3D-printing is maintaining consistent quality of layer-to-layer adhesion, and on the larger scale, homogeneity of material inside the whole printed object. An approach for mitigating and/or resolving those problems, based on the rapid and reliable control of the extruded material temperature during the printing process, was proposed. High frequency induction heating of the nozzle with a minimum mass (<1 g) was used. To ensure the required dynamic characteristics of heating and cooling processes in a high power (peak power > 300 W) heating system, an indirect (eddy current) temperature measurement method was proposed. It is based on dynamic analysis over various temperature-dependent parameters directly in the process of heating. To ensure better temperature measurement accuracy, a series-parallel resonant circuit containing an induction heating coil, an approach of desired signal detection, algorithms for digital signal processing and a regression model that determines the dependence of the desired signal on temperature and magnetic field strength were proposed. The testbed system designed to confirm the results of the conducted research showed the effectiveness of the proposed indirect measurement method. With an accuracy of ±3 °C, the measurement time is 20 ms in the operating temperature range from 50 to 350 °C. The designed temperature control system based on an indirect measurement method will provide high mechanical properties and consistent quality of printed objects.

## 1. Introduction

Presently, methods of additive manufacturing or 3D-printing find their application in architecture, construction, automotive and aerospace, clothes manufacturing and even in food production. It is fair to say that by now, a number of additive manufacturing technologies have reached the stage when their application in the industrial production of functional products has become possible [1,2,3,4].

Compared to subtractive manufacturing, 3D-printing has a number of major advantages. Namely: material cost reduction [5,6,7,8,9,10] (up to 90%), manufacturing cycle time reduction [8,11,12,13] because of rapid prototyping or simplification of the technological process, the opportunity to produce one-piece constructions with a complex shape. Together with the rapid development pace of generative design technologies, additive manufacturing allows one to provide significant reduction in the weight of a vehicle, products made of expensive materials, etc. [8,9,10,13,14,15,16,17].

It is worth noting that by now, there is an entire industry devoted to the production of prostheses, implants, stimulators, artificial organs and scaffolds from biocompatible materials. One of the most promising trends in this area is an application of modern high-tech polymer materials or polymer matrix composites. At the same time, using such materials in a biomedical application presents a number of problems: namely, bioinertity/bioactivity of the material, cell adhesive ability and the complex shape of tissue engineering scaffolds, etc.

Presently, additive manufacturing is being actively researched and implemented in medicine. Additive manufacturing technologies allow the production of polymer prosthesis, implants, scaffolds, etc., with a required shape and internal structure [18,19,20,21,22,23,24].

In recent years, a huge amount of research effort has been devoted to studying polyetheretherketone(PEEK) and its application in the biomedical field [25,26,27,28,29,30,31,32]. Three-dimensional printing of PEEK is carried out via fused deposition modeling (FDM) or fused filament fabrication (FFF) and associated with significant challenges. The high melting temperature and semi crystalline nature of PEEK make it very sensitive to varying processing conditions during the printing process. Both a high temperature of extrusion (about 440 °C) and consistent quality of layer-to-layer adhesion are needed in order to achieve the required mechanical properties of the printed object.

Fused deposition modeling is the most widespread 3D printing technology [33,34] and was created in 1986 by S. Scott Crump.

The essence of the technology is in building an object based on a digital model by depositing molten polymer material layer by layer on the platform through a heated nozzle [35,36].

One of the biggest challenges of FDM/FFF 3D printing is the dependence of layer-to-layer adhesion quality on bonding temperature: a function of the extruded material temperature and the bed temperature (or temperature of the already deposited layers of a printed object). 

This problem is especially critical for 3D-printing with high-performance materials (PEEK, PEI, etc.) because a change in this combination of temperatures by only a few degrees leads to a drop in quality (mechanical properties, external appearance, etc.).

Bonding temperature is a function of the heat input into already deposited layers of printed object (that depends on the trajectory of the extruder and the time spent by the extruder in the certain areas of the deposited layer of the printed object) and the heat input due to new extruded molten material.

The uniformity of the heat input may be achieved by compensating for the differences in the heat input in different areas of the already deposited layers of the printed object by a corresponding change in the heat input directly during the printing process.

A special case of such compensation is an increase in the layer time (reducing a printing speed and, consequently, extrusion speed), an increase in the cooling rate of the already deposited layers of the printed object via forced airflow, a temperature change of the already deposited layers of the printed object (slow change in the bed temperature). The listed capabilities are available in the existing (accessible) software in the field of FDM 3D printing (3D slicers). However, 3D slicers cannot purposefully provide uniformity of the heat input when planning a trajectory.

At the same time, conventional extruder for FDM 3D printer does not allow rapid changes in an extrusion temperature of the material during the printing process (due to significant mass of the hotend assembly, as well as the low power resistive heater), which prevents the setting of the required bonding temperature at certain points (segments) of the trajectory (the certain areas of the deposited layer of the printed object).

In general, the conventional extruder for an FDM 3D printer (for example, E3D V6, E3D Volcano or E3D Lite 6, Great Britain) consists of a hotend assembly (nozzle+heating block) or (hot) part that melts the polymer material and a (cold) part that feeds the solid polymer material (filament) into the hot part [37]. Most of the existing FDM printers employ indirect resistive heating of the nozzle [38]. At the same time, such systems are characterized by a big mass of the hotend assembly (from 20 g), low temperature limit (up to 260–300 °C), low power (~70 W) and sensitive to sudden temperature changes heating elements [39,40,41,42,43].

As temperature sensors in conventional extruders for FDM 3D printing are used thermistors or thermocouples. Due to the design features of the described extruder, the set temperature is provided only at the installation location of the temperature sensor. The temperature sensor is installed in heating block far away from the nozzle and extruding material. Hence, the sensor provides point measurement of hotend assembly temperature far away from the resistive heating element and the nozzle. This results in uneven heating of the nozzle and the extruded material (up to 20 °C) [43,44]. 

The time constant of the sensors (thermistors, thermocouples) is of 100–140 ms [45]. Consequently, with ideal thermal contact between heating block (or nozzle) and the sensor, it takes five-time constants for the thermocouple or thermistor to respond to almost 100% of the total step change temperature (500–750 ms). However, when installing the sensor in the heating block of the conventional extruder, the thermal contact is far from ideal due to air gaps, poor quality thermal grease, etc. 

As a summary, when using thermoelectric sensors (thermistors, thermocouples), the nozzle temperature measurement accuracy of 5–15 °C [44,46,47,48]. 

Another available method is non-contact infrared pyrometers that enable one to measure the temperature with higher time resolution (250 ms) than thermoelectric sensors (500–750 ms) used in FDM 3D printing. Herewith, accuracy of its measurement depends on the emissivity and purity of the nozzle surface. However, the emissivity of the metal nozzle changes when heated up and the surface gets contaminated quickly during the printing process. For that reason, the application of these devices for measuring the temperature of the nozzle in commercial FDM 3D printers is currently unknown. However, infrared cameras are widely used in laboratory research to analyze the spatial and temporal distribution of temperature in the printed objects.

Both the flow velocity of the extruded material and the movement speed of the extruder vary during the printing process. This results in significant deviations of molten material temperature from the required one.

As the result of the analysis, the drawbacks of the conventional extruder for an FDM 3D printer were revealed:

1. High thermal inertia (lag) of the hotend assembly (nozzle+heating block) does not allow rapid temperature regulation of the nozzle during printing;
2. Design features of the conventional extruder and drawbacks of the described temperature control methods do not allow the provision of fast and precise temperature control of the extruded material;
3. There is uneven heating of the nozzle and the extruded material (up to 20 °C) [43,46].

Thus, the conventional extruder for an FDM 3D printer does not allow one to provide consistent quality of layer-to-layer adhesion [47,49,50,51,52,53,54,55,56,57,58,59,60], and on the larger scale, homogeneity of material inside the whole printed object. Particularly, usage of high temperature engineering thermoplastics (PEEK, etc.) and thermoplastic composites make no sense due to a lot of inner defects in the resulting printed object [55,56,57,58,59,60,61].

An approach for mitigating and/or resolving those problems, based on the rapid and reliable control of the extruded material temperature during the printing process, is proposed. This approach is based on the method of high frequency induction heating of the low weight nozzle (<1 g).

This is the method of direct, non-contact electric heating and, in the general case, allows heating of the exact area of a heated part surface to higher temperatures within a short period of time and more efficiently than indirect resistive heating [62]. These features enable one to isolate the mass of the heater from the mass of the heated element.

Usage of induction heating in FDM/FFF 3D manufacturing was described in detail in our previous article [63]. A multiphysics FEM model including electromagnetic and thermal problems for the proposed nozzle and inductor configuration was formulated, and numerically solved using COMSOL 5.2a. The optimal inductor shape and heating frequency were obtained during the parametric optimization step. The advantages of induction heating over the indirect resistive heating method were discovered; for example:

1. rapid heating of the nozzle due to its isolation from the high power induction heater (peak power > 300 W);
2. rapid cooling of the nozzle due to its low mass;
3. uniform heating of the nozzle and extruded material due to optimization of the induction heating frequency and geometric shape of the inductor.

However, despite the obvious advantages, the described solution has a significant problem. To ensure the required dynamic characteristics of heating and cooling of the low weigh nozzle in a high power heating system, a fast and reliable temperature measurement is needed.

The lack of fast temperature measurement methods for the low weight nozzle was one of the major technological hurdles to the efficient application of induction heating in FDM 3D manufacturing.

A state of the art review shows a wide spread of techniques that provide either precise or fast temperature measurement of objects with low mass [64,65,66]. Most interesting, however, is the eddy-current (resonance) method for temperature measurement of pots made of ferromagnetic alloys presented in the article and patented by a group of researchers from the University of Zaragoza [67,68]. It is based on measurement of current frequency in a resonant circuit containing inductor. The frequency varies with the temperature-dependent parameters of the pot. This method is intended for temperature control of objects with high mass and there is no way to measure and heat at the same time.

The possibility of using other methods (thermoelectric, pyrometric) depends on solving all the mentioned problems, and mitigating interference of the strong alternating electromagnetic field on the measurement device and its wiring (for the wired ones) [69,70].

Eventually, the known prototypes of the extruder for FDM 3D manufacturing using induction heating of the nozzle have significant thermal inertia and do not differ substantially from conventional extruders [71,72,73,74,75,76,77].

Excess temperature of the nozzle may lead to both its permanent deformation and burnout of the polymer material. On the other hand, a high cooling rate of the nozzle during material extrusion (up to 20 °C/s at an extrusion speed of 40 mm/s) may lead to disruption or termination of the extrusion process.

Other optimization methods/techniques or tools of the FDM 3D printing process listed in [2]. The existing optimization methods of the FDM 3D printing process do not allow controlling the heat input during the printing (extrusion) process and maintaining consistent quality of layer-to-layer adhesion, and on the larger scale—homogeneity of the material in whole printing object. 

## 2. Materials and Methods

### 2.1. Temperature Measurement Technique for the Ferromagnetic Nozzle

To ensure the required dynamic characteristics of heating and cooling processes in a high power induction heating system, an indirect (eddy-current) temperature measurement method is proposed. It is based on the application of ferromagnetic alloys with the required mechanical properties as the nozzle material and on dynamic analysis over various temperature-dependent parameters during the heating process. One such parameter, the magnetic permeability of iron, directly depends on the temperature and was previously used for indirect temperature measurement.

### 2.2. Testbed System

This kind of temperature control loop consists of an induction heating coil, low weight nozzle (<1 g) and high power temperature controller (peak power > 300 W) and requires fast and precise temperature measurement.

A method for fast measuring of temperature-dependent parameters during heating is proposed. To implement this method in the control system and achieve high accuracy temperature measurement, it is necessary to determine the dependence of the electrical properties of the nozzle material (resonant circuit parameters) on the temperature.

To determine such dependence, a testbed system was designed, which comprises laboratory power supplies with U = 24V, Imax = 20A, a DRV8302-based power controller, a high frequency (HF) voltage source inverter using power MOSFETs, and a Control Board based on an ARM-microcontroller STM32F334R8 by STMicroelectronics. Rated values of elements in the inductor-capacitor-inductor (LCL)-resonant circuit were converted to fit the predetermined operating frequency of f = 120 kHz. The induction heating coil of a specified diameter and height was formed with 20 coils of copper high-frequency litz wire (d = 0.75 mm, 2 layers).

**Figure 1:**
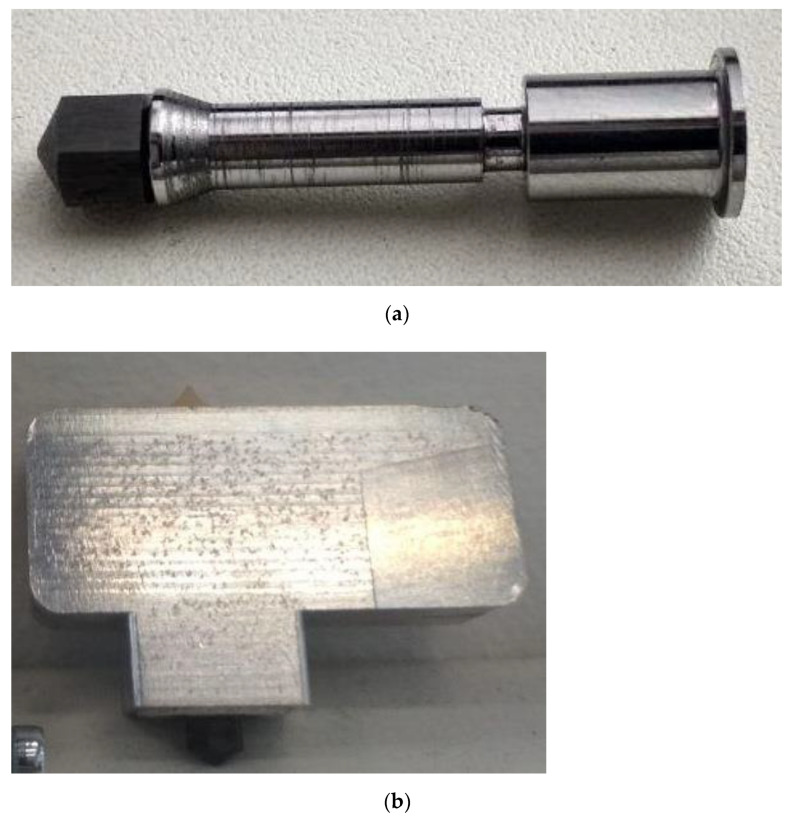
(**a**) Appearance of the ferromagnetic nozzle, (**b**) appearance of the pilot extruder.

The appearances of the ferromagnetic nozzle as well as the pilot extruder are shown in Figure 1a,b.

**Figure 2:**
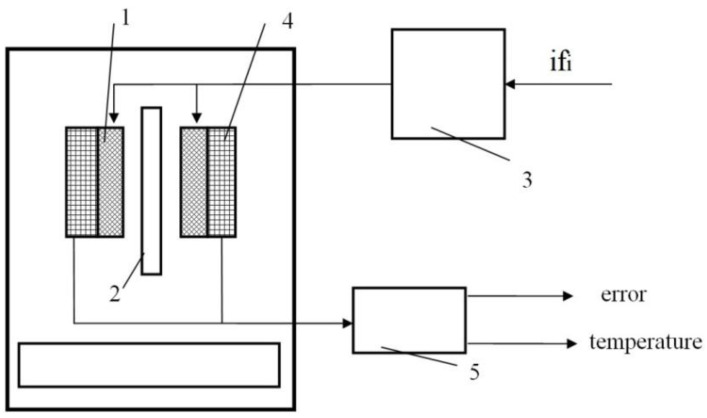
Functional block diagram of the testbed system: (**1**) the induction heating coil; (**2**) the nozzle; (**3**) high frequency (HF) inverter; (**4**) the sensing coil; (**5**) the unit for recording and processing a measuring signal (ARM-microcontroller).

A functional block diagram of the testbed system is shown in Figure 2. if_i_ is the control signal, “error” is the measured signal amplitude, temperature is the nozzle temperature at the beginning and at the end of the experiment.

An induction heating coil (1) was installed inside an aluminum cylindrical mandrel that functions as a shield against the high frequency magnetic field, and as a mechanical fastening of the heater to the extruder frame, a magnetic flux concentrator made of supermalloy in the form of an external cylindrical shell of the inductor was used. Inside the induction heating coil, (1) the nozzle (2) for FDM 3D manufacturing of a preset configuration made of the ferromagnetic alloy AISI430 was installed. The inductive load was extended to an LCL series-parallel resonant circuit by adding a further capacitive device in parallel with the induction heating coil (1) and inductive device in series with the parallel resonant circuit. The LCL resonant circuit was supplied by a high frequency phase-shifted full bridge inverter (3).

In phase-shift modulated (PSM) control, the two transistors of each diagonal switch are operated at nearly 50% duty-ratio. The output of each diagonal switch pulsates between V_in_ (power supply voltage) and 0. The length of the zero intervals is controlled by phase-shifting the two diagonal switch outputs with respect to each other. During zero intervals, either both transistors at the top, or both transistors at the bottom are on, creating a short circuit across the primary winding. The gate voltage of the transistors on the top is controlled by the two direct signals Q1 and Q2, while the gate voltage of the transistors on the bottom is controlled by the corresponding inverted signals. The amount of phase shift (if_i_) between the diagonal switches (between Q1 and Q2) decides the amount (percent) of power consumption [78,79]. In this example, the phase shift range from 0 to 180° between Q1 and Q2 signals corresponds to a range of 0 to 45,000 in terms of processor ticks.

**Figure 3:**
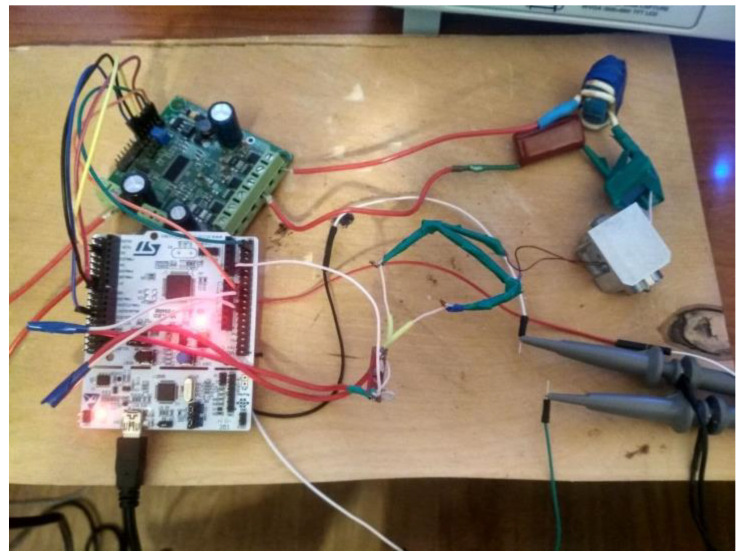
Appearance of the testbed system.

The inductor (1) current varies with the resonant circuit parameters during heating of the nozzle (2). To register such changes (the desired signal), the secondary winding was added to the induction heating coil (1) as the sensing coil (4). The outputs of the secondary winding are indirectly connected to a microcontroller analog input (5). The measured signal amplitude is expressed as a dimensionless value “error” as a result of digital signal processing. Nozzle (2) temperature at the beginning and at the end of the experiment was measured by a contact type thermometer UT325. The appearance of the testbed is shown in Figure 3.

### 2.3. Basics of an Indirect (Eddy-Current) Temperature Measurement Method

**Figure 4:**
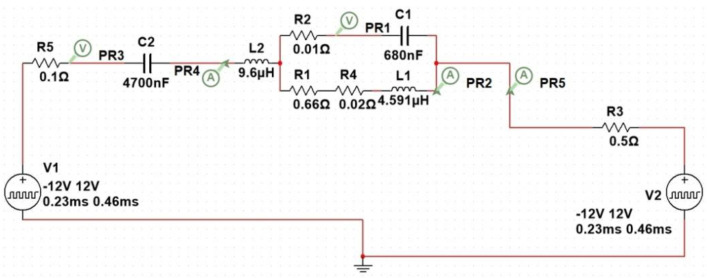
Multisim model of LCL series-parallel resonant circuit.

For implementation of the proposed method, LCL topology for high power induction heating was used. The LCL resonant circuit was designed using a Mutlisim simulation environment according to Figure 4. Voltage sources V1 and V2 operate simultaneously and are 180 degrees out of phase with each other.

The simple equivalent circuit describing the heating induction coil and the nozzle consists of a coil (L1) and a resistor (R1) connected in series. The power factor is improved because of an additional capacitor (C1) and inductor (L2) in the circuit. The purpose of the inductor L2 is to match the impedance of a source and that of its load, while working close to the resonant frequency. It is also provides negligible switching losses due to operation above resonance (ZVS).

For a resonant circuit with a high quality factor Q, the approximation (1) is valid for the resonance point.

(1) $$\frac{I_{1}}{I_{2}}=\frac{L_{2}}{L_{1}},$$

where *I*_1_ is the inductor (*L*_1_) current and *I*_2_ is the inductor (*L*_2_) current.

The LCL topology provides a load-independent output voltage [80,81]. It should be noted that the natural (resonance) frequency of the parallel resonant circuit L1C1 is independent of other electrical circuit elements. The higher the quality factor Q, the lower the impact. Therefore, both the natural frequency of the parallel resonant circuit L1C1 and the inductor (L1) current amplitude vary only with the electrical properties of the nozzle and the induction coil during the heating process. During heating of the nozzle, the operating frequency was set close to the resonance frequency f = 120 kHz.

The measurement method is based on the dependence of the electrical conductivity and the magnetic permeability of the material of the nozzle with respect to its temperature. The operating temperature range is from 20 to 1100 °C because the Curie temperature of the ferromagnetic alloy used as the nozzle material is about 1150 °C.

When a ferromagnetic material is heating by induction, the heat generation also occurs due to hysteresis losses. However, this effect rapidly abrupts as temperatures approach the Curie point, after which the material becomes completely non-magnetic [82]. The change in the electrical conductivity and magnetic permeability of the material leads to a change in the electrical equivalent impedance of the parallel resonant circuit L1C1. Variation of the load impedance does change both the input current consumed and the resonance frequency. The inductor (L1) current amplitude also depends on power consumption (output voltage). Output voltage varies with the desired nozzle temperature, the flow velocity of the extruded material or during temperature regulation.

From the change of inductor (L1) current amplitude during experiments, it is possible to determine its dependence on the nozzle temperature and power consumption.

A significant deviation of the operating frequency from the resonance frequency results in an inductor (L1) current decrease and an unacceptable drop in heating efficiency. In this case, it is necessary to determine the frequency range that corresponds to the entire operating temperature range. Resonance frequency must be set by adjusting the values of the electronic components in such a way that deviation from it during heating of the nozzle should not lead to a significant change in current amplitude. Besides, a slightly inductive load is desired to provide ZVS operation. A frequency meter can be used for calibration. However, for the LCL resonant circuit with a high quality factor Q, the required frequency range is very narrow (about 2000 Hz). It is desired to use a phase meter for more accurate calibration. Such a small deviation of the resonance frequency from the predetermined operating frequency of f = 120 kHz results in a minimum phase shift between the inductor (L1) current and output voltage. From the point of view of the described testbed system, it is correct for the operating temperature range from 20 to 500 °C in which the extrusion process of almost all existing filaments for FDM 3D-manufacturing occur.

**Figure 5:**
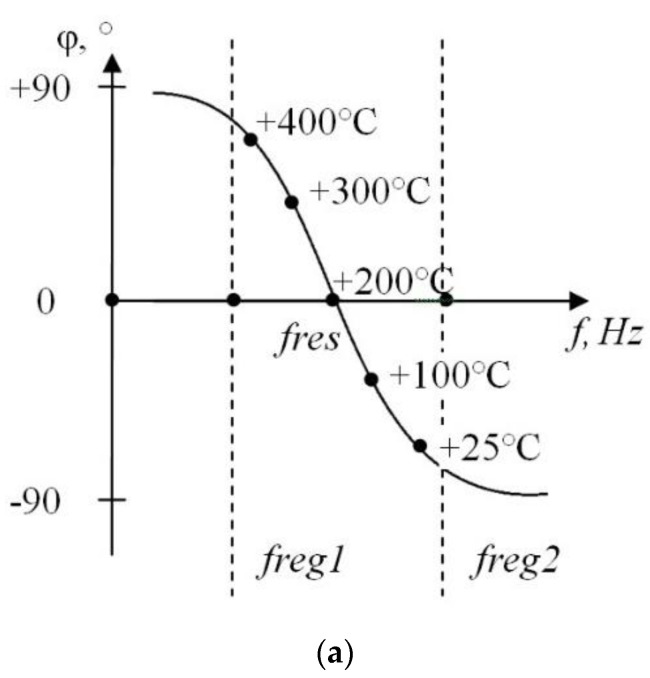

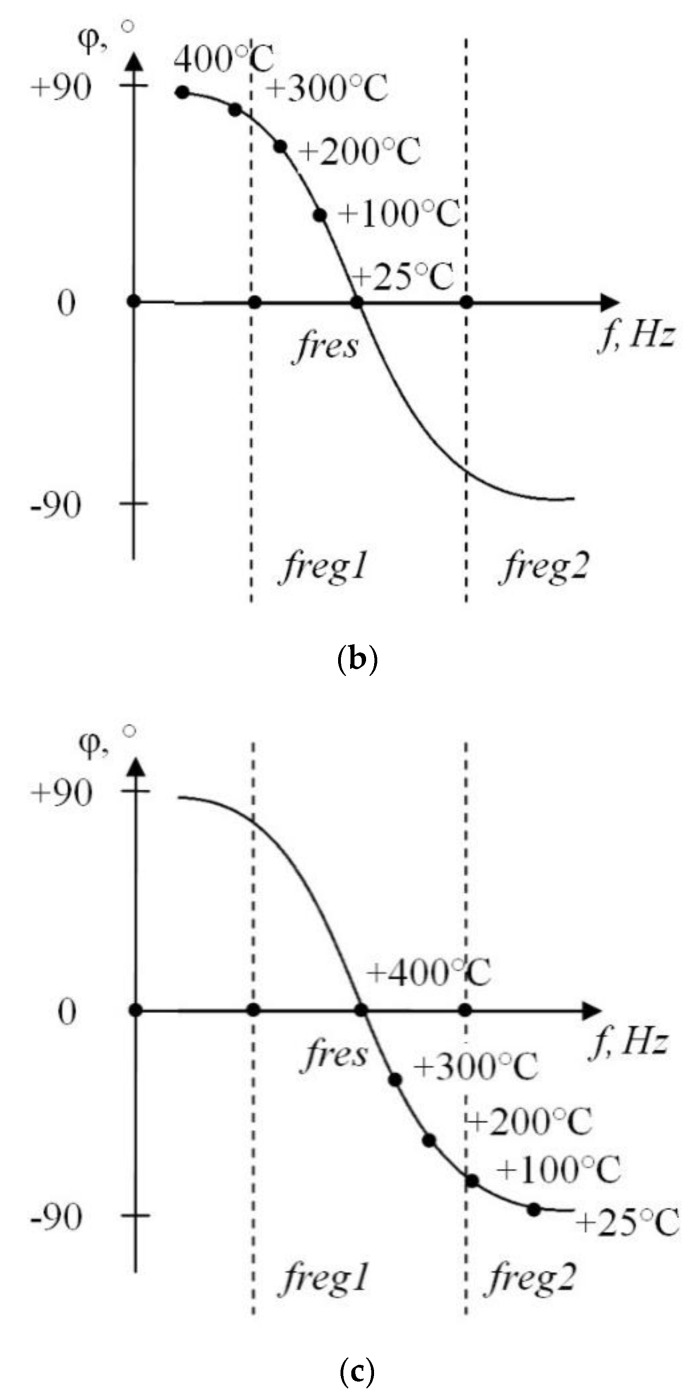
Frequency range and corresponding phase shift between inductor (L1) current and output voltage. Nozzle temperature (T) for resonance condition. (**a**) T = 200 °C, (**b**) T = 25 °C, (**c**) T = 400 °C.

The phase frequency characteristic is shown in Figure 5: fres is the resonance frequency of the parallel resonant circuit, freg1…freg2 is the frequency range in which inductor (L1) current amplitude is measured, T is the nozzle temperature.

The inductor (L1) current varies with the resonant circuit parameters during heating of the nozzle. The sensing coil (L3) as the secondary winding was added to obtain the desired signal. A varying current in the induction heating coil produces a varying magnetic field which induces a varying electromotive force across the sensing coil: thus, electrical energy is transferred between electrically isolated coils. In this example, the sensing coil has a 10 mm inner diameter, 15 mm outer diameter, 16 mm height coil. The sensing coil was formed with 20 coils (2 layers).

**Figure 6:**
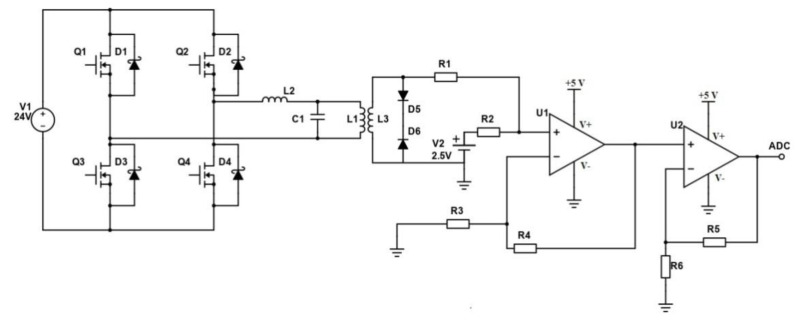
High-frequency induction heating system with LCL-resonant output and Sensing circuit.

To improve the accuracy of a measurement, the outputs of the secondary winding were connected to an operational amplifier (U2) with a gain of 40. The diode clipper (D5, D6) with clipping limits of ±0,5V cuts off both halves together of the signal waveform. The bipolar desired signal converts to unipolar to drive an analog-to-digital converter (ADC) via summing amplifier (U1) with a gain of 5 [83]. Thus, a significantly amplified desired signal is measured by the ADC. The high-frequency induction heating system with LCL-resonant output and Sensing circuit are shown in Figure 6. In this example: D1, D2, D3, D4—intrinsic antiparallel diodes, R1 = 1 kΩ, R2 = 5 kΩ, R3 = 1 kΩ, R4 = 5 kΩ, R5 = 40 kΩ, R6 = 1 kΩ.

To measure the amplitude of the desired signal, PSM and ADC were synchronized to an operation of a high-resolution timer. Thus, the synchronization signal triggers ADC conversions at the same time as the signal Q1 is generated. Herewith, the output voltage is in phase with the signal Q1. And the inductor (L1) current is in phase with output voltage when the load is resistive (resonance condition). Therefore, there is a varying phase shift (above mentioned) between desired signal and signal Q1 (fixed moment of an ADC conversion) during heating process of the nozzle. So, the measured amplitude of the desired signal also depends on this phase shift. In this example, the 12-bit ADC is used. Consequently, the ADC converts 0 to5V on its input into dimensionless value in the range from 0 to 4095.

As can be seen, the operating temperature range from 20 to 500 °C should corresponds to the phase shift range from −90° to +90° between desired signal and output voltage (Figure 5a). Measurements are made once per desired signal period and limited by the operating frequency of f = 120 kHz.

The extrusion temperature of the most commonly used materials in the FDM 3D manufacturing is above 200 °C, for example, polylactic acid (PLA), acrylonitrile butadiene styrene (ABS), styrene-butadiene-styrene (SBS), polyethylene terephthalate (PET), polypropylene (PP), polyamide (PA), polyetherimide (PEI), polyetherketoneketone (PEKK), PEEK, etc. Therefore, operation above resonance frequency is provided. Inductive load generates parasitic oscillating voltage during the switching of MOSFETs, while stray inductances are included in the series inductor L2.

To reduce such noise in the circuit as well as achieve better accuracy of the measurement, both oversampling and an exponential moving average filter were used. The dimensionless value “error” was obtained as a result of digital signal processing.

Oversampling and averaging is a method for improving ADC resolution. In this example, ADC resolution was increased from 12 bits to 17 bits [84,85]. Sampling frequencies above Nyquist frequency are called oversampling. It is sufficient to use a converter that can run at 1024 times the target sampling rate. Summing 1024 consecutive 20-bit samples can increase the SNR, effectively adding 5 bits to the resolution and producing a single sample with 17-bit resolution.

For each additional bit of resolution, the signal must be oversampled by a factor of four according to Equation (2):

(2) $$f_{p}=\frac{f_{n}}{4^{y}},$$

where *y* is the number of additional bits of resolution desired, *f_p_* is the sampling frequency requirement or Nyquist frequency, and *f_n_* is the oversampling frequency.

The number of samples required to get n bits of additional data precision is 4^*y*^. To get the mean sample scaled up to an integer with n additional bits, the sum of 4^y^ samples is divided by 2^y^.

This averaging is only effective if the desired signal contains white noise. The noise amplitude must be sufficient to cause the desired signal to change from sample to sample at least between two adjacent levels of quantization.

An exponential moving average (EMA) filter is used for smoothing a stream of data after oversampling and averaging [86]. An EMA filter allows you to specify the weight of the last measuring versus the previous filtered value, by setting the alpha parameter. The equations for an exponential moving average filter are:

(3) error=xprev×(1−alpha)+xnew×alpha,

(4) xprev=error,

where *xnew* is the current filter input value, *xprev* is the previous filter output value, “error” is the current filter output value after the last measuring, alpha is the smoothing factor in the range from 0 to 1. The higher the value, the less smoothing (the higher the latest measuring impact). If *alpha* = 1, the output is just equal to the input, and no filtering takes place.

Notice that the calculation does not require the storage of past values of “error” and only the previous value, which makes this filter microcontroller memory friendly.

The main factors, defining the total measurement delay of the proposed device, are the ADC sampling delay (2Tc–61Tc, where Tc is the frequency of the ADC peripheral module of the system on a chip (SoC) in use, Tc = 1/500 kHz), and the delay of the oversampling process used to extend the measurement resolution. Thus, the total delay depends on the measurement device desired output resolution and varies from 10 µs (for the native 12-bit resolution of the ADC, and no oversampling) for the 8 °C temperature resolution, up to 100 ms (for the 20-bit oversampled output), and 0.05 °C resolution. Therefore it needs to be stated, that the maximum sampling frequency in the proposed method is limited by the heating frequency (120 kHz), cause successful measurement is possible only in close proximity to the zero-crossing moment of the current in the inductor circuit.

## 3. Results and Discussion

### 3.1. Desired Signal Amplitude Measurement

A series of experiments was carried out using the described testbed system with different fixed output voltage (power consumption in the range from 0 to 100%). The nozzle was heated from 25 °C to Curie temperature. Then, the nozzle was cooled to an ambient temperature and after that, heated again from 25 to 750 °C in each experiment. In this example, a nozzle temperature of 25 °C corresponds to “error” = 0. The nozzle temperature at the beginning and at the end of the experiment was measured by a contact type thermometer UT325. The desired signal amplitude (error) was continuously registered during the heating process.

**Figure 7:**
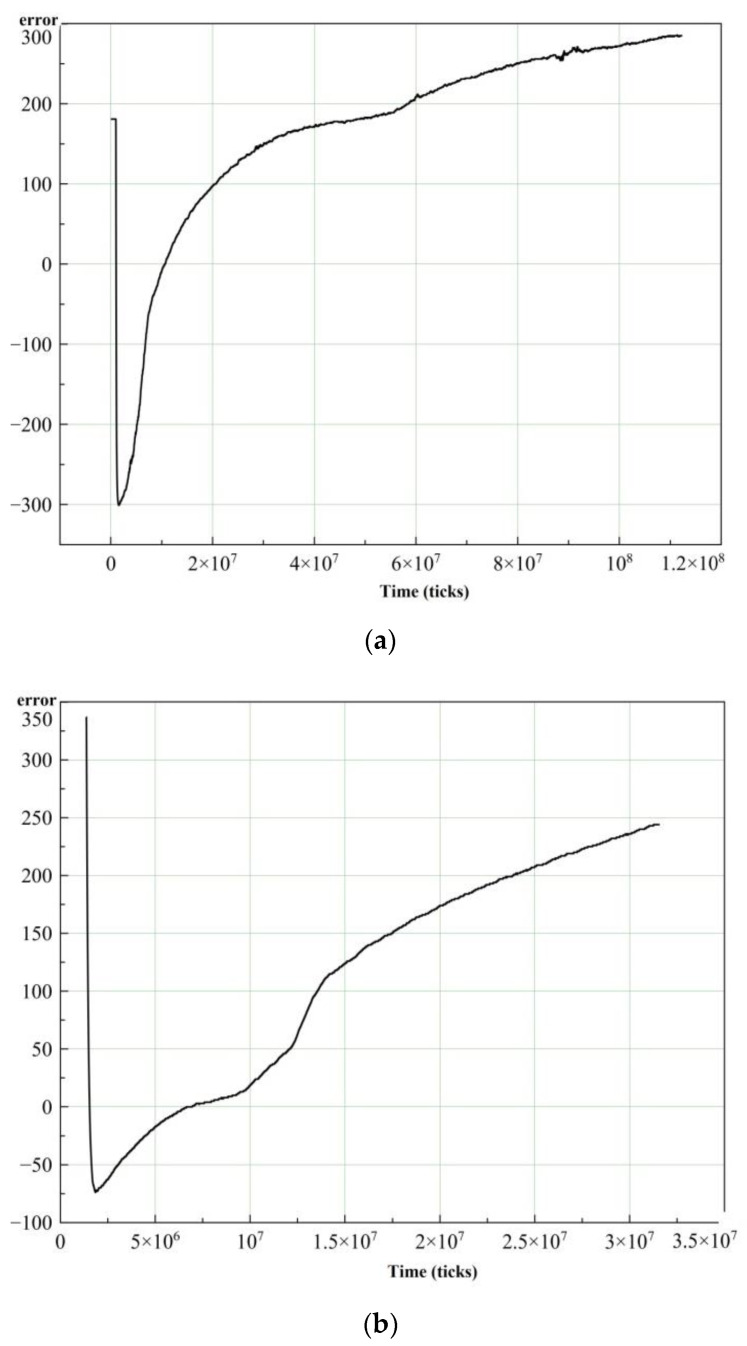
Desired signal amplitude (error) as a function of time (ticks): (**a**) power consumption of 80%, (**b**) power consumption of 90%.

On the basis of the measurement results, the desired signal amplitude (error) was obtained as a function of time (in processor ticks monitored by debug software). This dataset was obtained for a fixed power consumption in 10% increments. The results of the experiments with power consumption of 80% and 90% are shown in Figure 7. Figure 7 shows the dramatic decline of “error” at the very beginning of the charts. The reason for this is the nozzle temperature approaches the Curie point and the magnetic permeability of the ferromagnetic alloy drops down. Reduction of the magnetic permeability of the nozzle material leads to the resonance frequency rising above the operating frequency. It is worth noting that the graphs of the error(t) with distinctive power consumption are very different from each other. The reason is that the magnetic field strength of the coil directly depends on power consumption, and at the same time, magnetic permeability dependence on the temperature of the nozzle varies with the magnetic field strength. Moreover, the dependence of “error” on time during the heating process of the nozzle is non-linear. Figure 7a shows that the nozzle temperature reached 750 °C on the fifteenth second of heating. Figure 7b shows the nozzle temperature reached 750 °C on the fifth second of heating.

To simplify further data processing, the number of points in the curves was reduced by three orders of magnitude via the Ramer–Douglas–Peucker algorithm [87]. The algorithm allows preserving of the shape of graphs, inflection points and extrema of functions. This algorithm was implemented in Microsoft Visual Studio 2017.

At the last stage, the number of points in the curves was manually reduced to 40.

**Figure 8:**
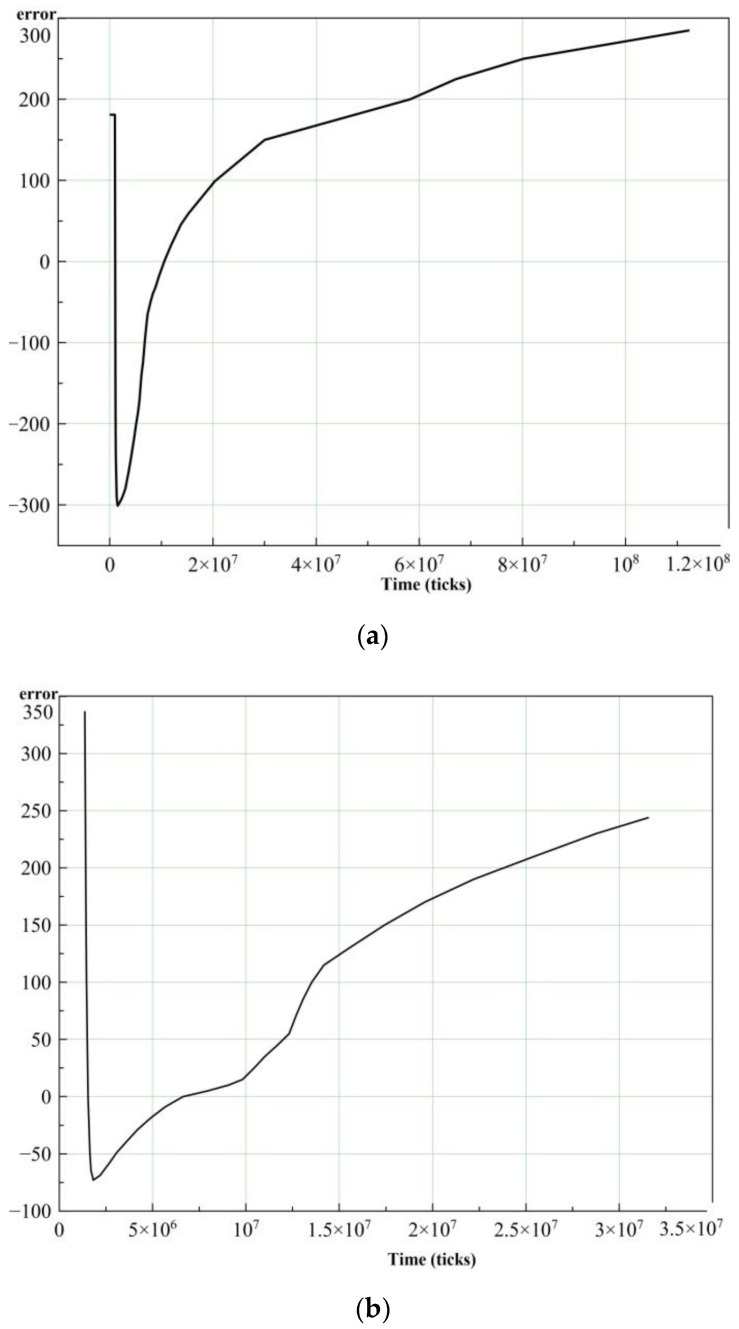
Desired signal amplitude (error) as a function of time (ticks) after filtration: (**a**) power consumption of 80%, (**b**) power consumption of90%.

The results of the experiments with power consumption of 80 and 90% after filtration are shown in Figure 8.

**Figure 9:**
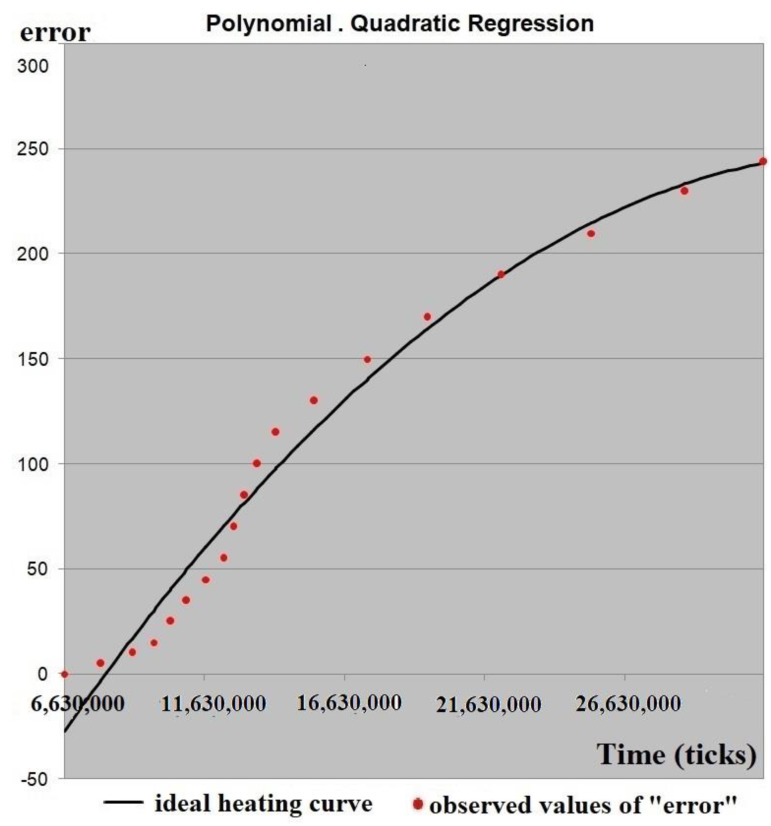
Ideal heating curve of the nozzle with power consumption of 90%.

The ideal heating curve of the nozzle was obtained by implementation of a quadratic regression model onto the dataset for power consumption of 90% as an example. The ideal heating curve of the nozzle is shown in Figure 9. For this fitting, a value of the coefficient of determination of R^2^ = 0.97 was obtained.

The regression model was validated by a contact type thermometer UT325. A thermocouple was placed inside the nozzle during the heating process without filament. The ideal heating curve is acceptable with respect to the real heating curve and almost repeats it with a slight time delay.

The range of the ideal heating curve (the graph of the estimated regression equation) was linear scaled to the range of the real heating curve (nozzle temperature at the beginning and at the end of the experiment in the range from 25 to 750 °C).

In this purpose, the coefficient of proportionality between the maximum temperature of the nozzle and the maximum “error” was found. Using this, coefficient value of the function (regression equation) in 10 °C increments were found and arguments (time points) of the function calculated. The nozzle temperature at certain time points was obtained. For the corresponding arguments (time points) of the function (regression equation), values of “error” were found from the original dataset (Figure 7).

Thus, correspondence between the nozzle temperature and “error” during the heating process (at certain time points) was determined for fixed power consumption in 10% increments.

### 3.2. Regression Model

At last, to provide accurate measurement of the nozzle temperature, statistical analysis of experimental data was performed and the dependence of the desired signal on temperature and magnetic field strength was determined.

Table 1 represents the results of data analysis. Headers row: power consumption range. Headers column: nozzle temperature range. Cells: observed values of «error».

Power consumption depends on the control signal or, rather, the amount of phase shift (if_i_) between diagonal switches (between Q1 and Q2), as already mentioned.

Thus, the amplitude of the desired signal expressed as dimensionless value «error» is the dependent variable. Nozzle temperature (T_i_) is the first independent variable and magnetic field strength expressed as phase shift (if_i_) is the second independent variable.

The regression equation was estimated using multiple nonlinear regression analysis. An F-test (ANOVA) was used to test the statistical significance of the overall relationship between the dependent variable and independent variables. A regression analysis was performed in PTC Mathcad 15.

The dependence of the «error» variable on two independent variables can be described by following the regression equation:

(5) $$Yield=A\times T_{i}+B\times if_{i}+AB\times T_{i}\times if_{i}+BB\times if_{i}{}^{2}+ABB\times T_{i}\times if_{i}{}^{2}+C,$$

with the following coefficients (Table 2).

The correlation coefficient is close to unity (0.999); thus, the relationship between dependent variable and independent variables is a rigorously functional one.

F_fact_ calculated from the data is equal to 1.2 × 10^5^. The critical value of F_crit_ = 3.01 determined from the tables is a function of the degrees of freedom and the significance level (α = 0.05). The regression equation yield (T_i_,if_i_) is statistically significant with reliability of 95% because F_fact_ is greater than the critical value F_crit_ of the F-distribution.

**Figure 10:**
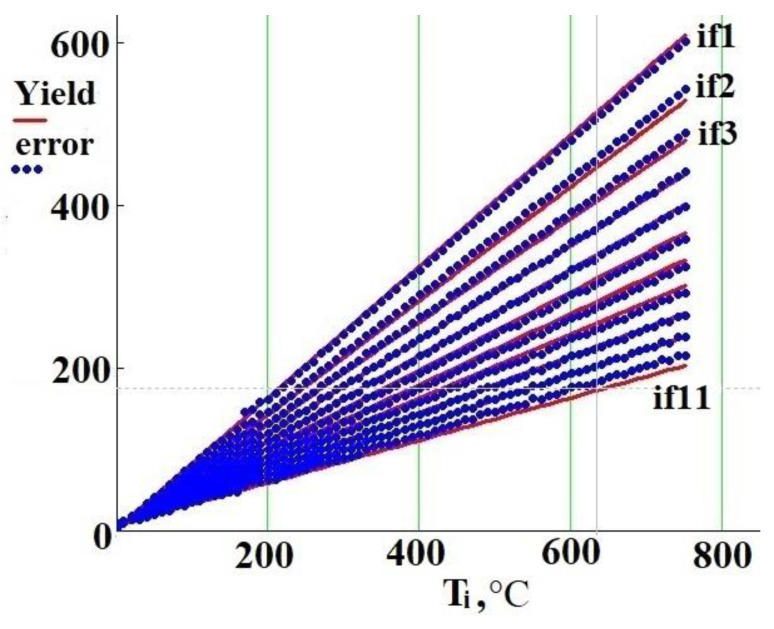
Estimated regression equation and observed values of “error”.

The graph of the estimated regression equation yield (T_i_,if_i_) and observed values of «error» are shown in Figure 10.

**Figure 11:**
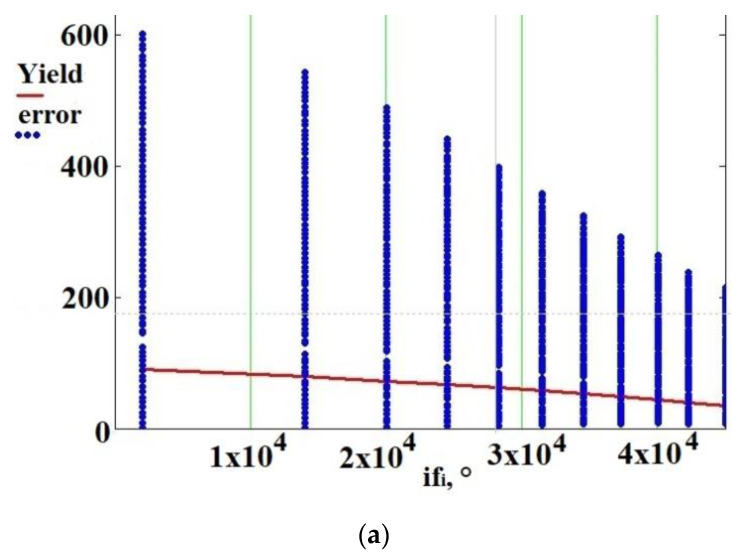

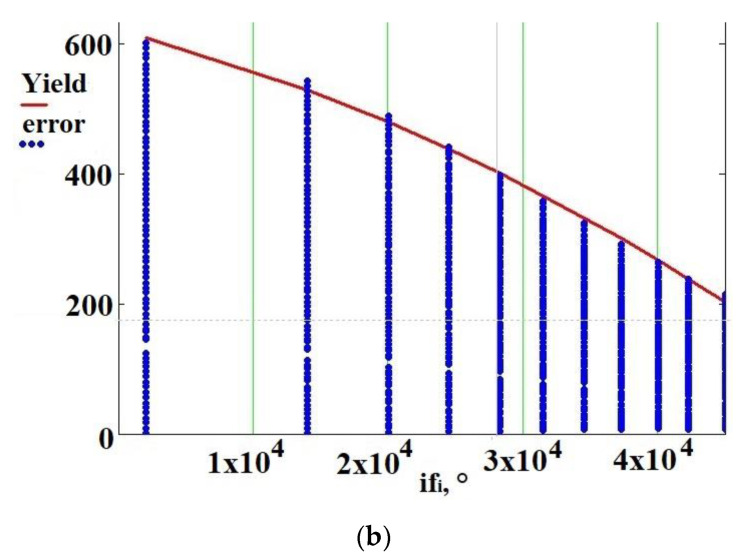
Desired signal amplitude (error) dependency on magnetic field strength (phase shift if_i_) with fixed nozzle temperature: (**a**) 110 °C, (**b**) 750 °C.

Figure 10 shows that increasing power consumption from 0 to 100% is accompanied by narrowing the «error» range by three times. When power consumption is of 0.2% (if_1_), then «error» is in the range from 0 to 600. When power consumption is of 100% (if_11_), then «error» is in the range from 0 to 215. Figure 11 shows the «error» dependency on if_i_ with fixed nozzle temperature. As can be seen, the dependence of «error» on if_i_ is non-linear. Moreover, the higher the temperature of the nozzle, the more significant the non-linearity. This figure also shows that the dependence of temperature on power consumption is non-linear.

The design system in steady-state provides operation in the mentioned temperature range from 20 to 500 °C with power consumption of 25–60W (0.7–5%) if it is a low extrusion speed (10 mm/s), and with power consumption of 35–85W (1.3–9%) if it is a high extrusion speed (100 mm/s).

**Figure 12:**
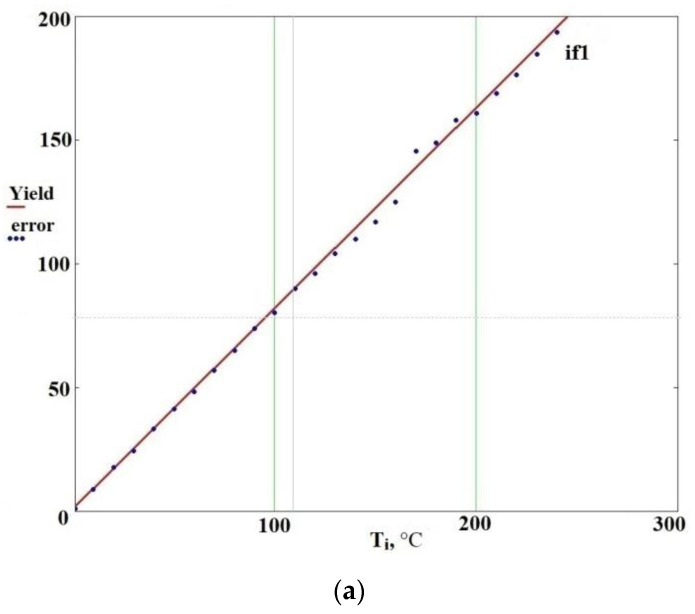

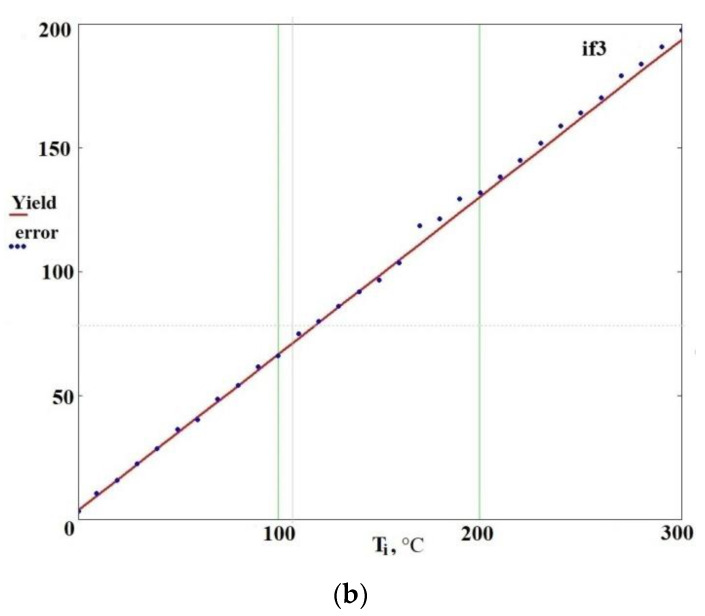
Observed versus predicted values of “error”. Dependence of “error” on temperature with fixed power consumption: (**a**) 0.2%, (**b**) 20%.

Figure 12 shows the non-linear dependence of «error» on temperature with fixed power consumption of 0.2 and 20%. The reason for this is non-linear dependence of the magnetic permeability of the nozzle material on temperature during the heating process, according to Figure 7. Figure 12 also shows observed versus predicted values of «error» and linear approximation of magnetic permeability dependence on temperature.

Application of the obtained regression model provides conversion from «error» to nozzle temperature (T) with high accuracy in the operating temperature range from 20 to 500 °C. Taking into account almost all described non-linear dependences, temperature measurement accuracy of ±3 °C during the heating process is provided.

### 3.3. Experimental Verification of Proposed Method

The proposed indirect (eddy current) temperature measurement method (sensing circuit) is used in the feedback loop. In other words, the proposed system is a closed-loop control system, as any temperature control system in existing FDM 3d-printers. 

Control system design (proportional-integral-derivative (PID) controller design, etc.) will be described in detail in the further publication of the results in journal with the corresponding topic.

The designed testbed system was integrated into an FDM 3D-printer to test its behavior in a real environment. A closed-loop temperature control system with non-optimal controller parameters were used during the calibration process and printing of samples.

Several products were made of PA and PEEK to demonstrate the possibility of using the proposed temperature control system in 3D-printing with high-performance materials.

Nylon (PA) and PEEK were chosen as the test materials, because these polymers are highly sensitive to the extrusion temperature. A temperature deviation of 5–10 degrees leads to a significant change in the characteristics of the printed product, a decrease in mechanical properties, changes in external appearance (product color, shape of the extruded line), as well as to the overheating of the material caused by uneven heating inside the nozzle or hotend assembly (which leads to the formation of visible bubbles in the extruded material).

**Figure 13:**
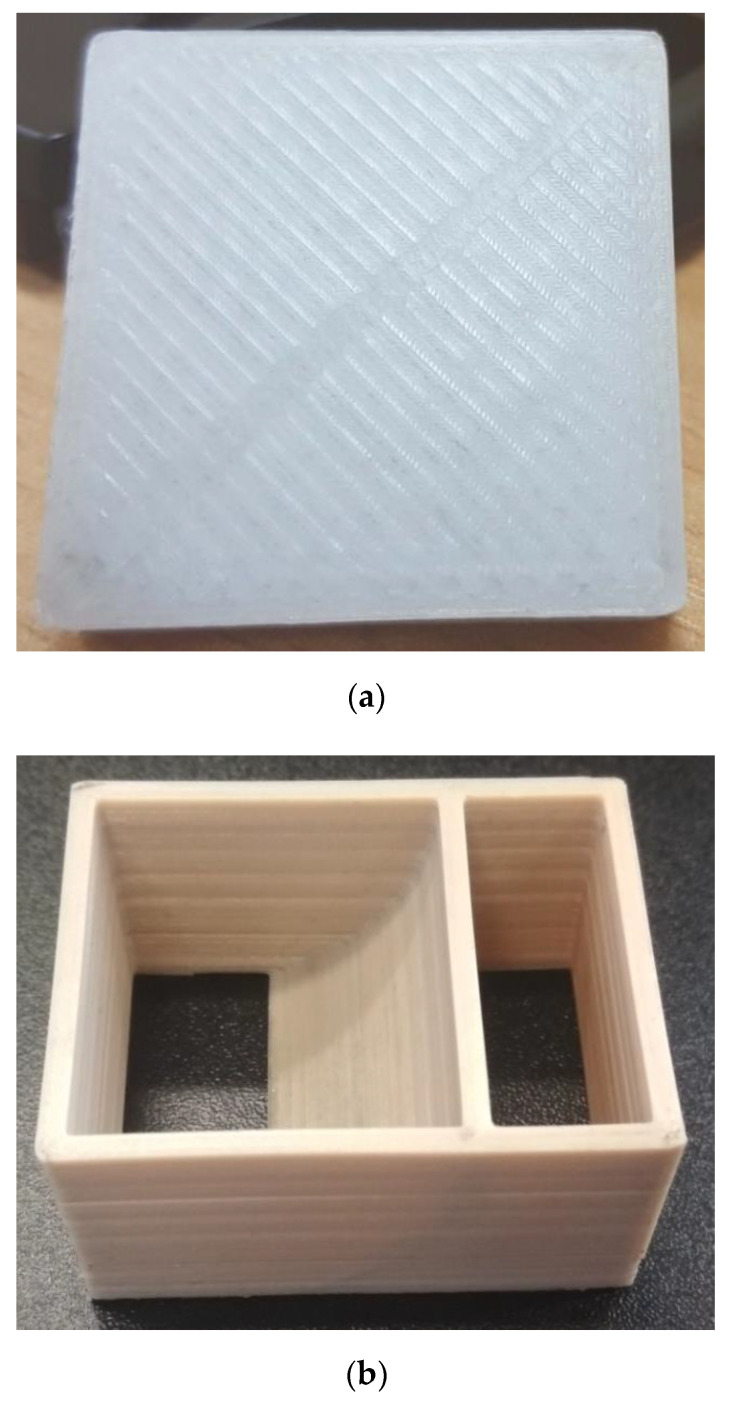
(**a**) Appearance of the test product surface printed of polyamide (PA); (**b**) appearance of the showpiece printed from polyetheretherketone (PEEK).

Several samples were printed of PA (box with a base of 30 × 30 mm and height of 5 mm). The printing output is shown in Figure 13a. All samples printed at an extrusion speed of 5 to 100 mm/s (for a nozzle of 0.4 mm) demonstrate an identical appearance.

Figure 13b shows the showpiece printed of PEEK (showpiece size: 50 × 30 × 25 mm). The figure shows the fairly uniform surface color of the showpiece printed of PEEK.

To investigate the influence of the proposed temperature control system based on indirect measurement method of mechanical properties and the quality of layer-to-layer adhesion of printing objects, several tensile specimens were made of ABS and PLA (specimen size: 115 × 25 × 2 mm). Samples were printed using 100% infill density with printing parameters as given in Table 3.

**Figure 14:**
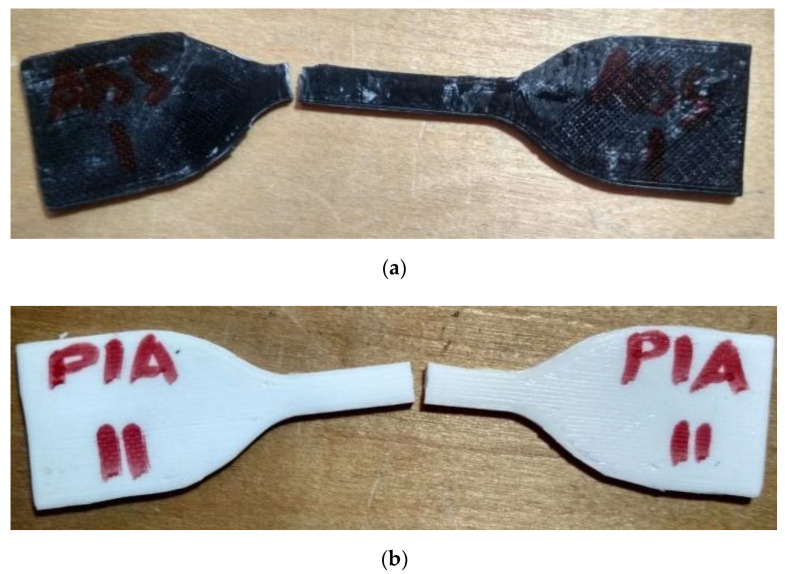
(**a**) Appearance of the fractured tensile specimen printed of acrylonitrile butadiene styrene (ABS); (**b**) appearance of the fractured tensile specimen printed of polylactic acid (PLA).

Tensile testing was carried out by using an electromechanical testing machine (Instron 5882). The testing speed for all specimens was 2 mm/min. Four specimens were tested for each batch and the testing was performed at ambient temperature (24 °C). Table 4 represents the resulting tensile strength values for all specimens. For example, the fractured tensile specimens ABS1 and PLA2 are shown in Figure 14.

**Figure 15:**
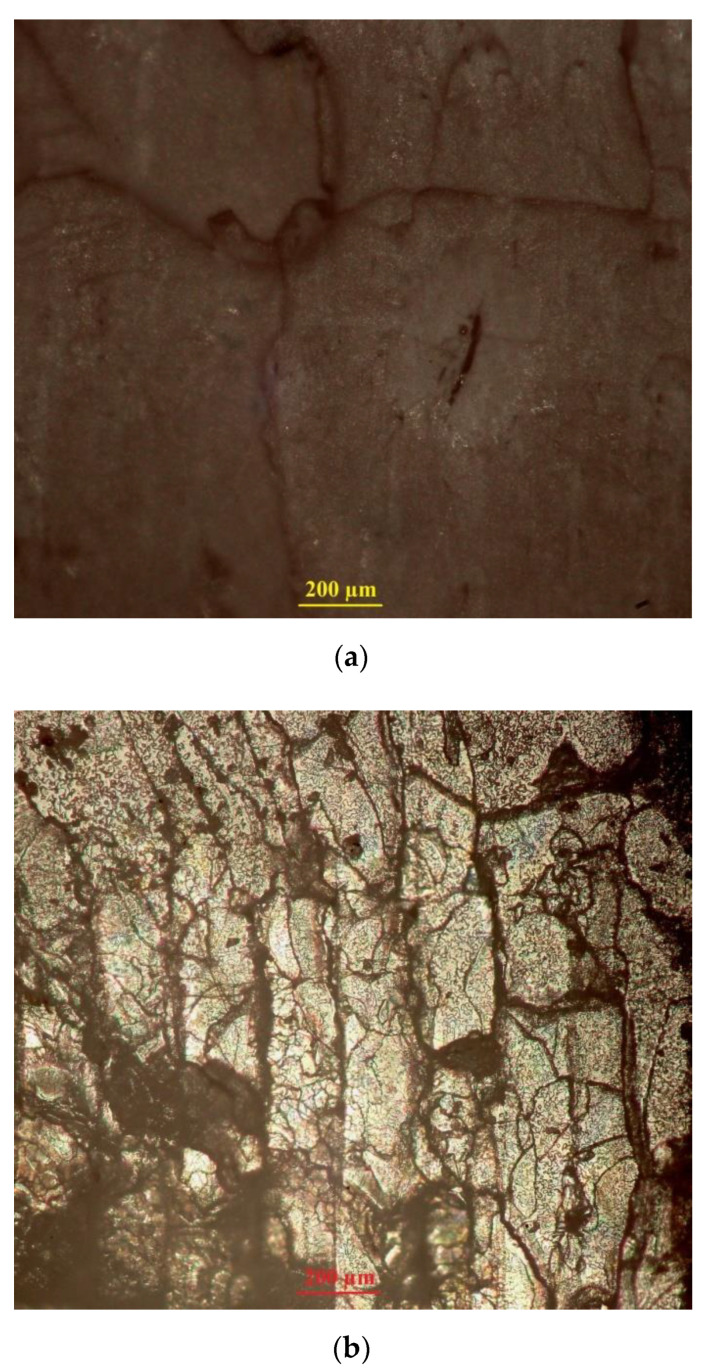
Optical microscopy of the cross-sectional surfaces of the FDM-printed specimens after tensile testing:(**a**) fractured tensile specimens printed of ABS; (**b**)fractured tensile specimens printed of PLA.

Figure 15 presents optical microscopy of the cross-sectional surfaces of the FDM-printed specimens (ABS1 and PLA2) after tensile testing. Photographs were taken perpendicular to the layers orientation using an Olympus optical microscope and microscope-specific camera.

As seen in the figure, there is little to no interlayer separation and splitting on the fracture surface of the printed tensile specimens. The tensile fracture of ABS1 and PLA2 specimens has are latively flat surface, indicating a good interlayer bonding strength in this case. In Figure 15a, there is one interlayer gap in the fracture surface of the tensile specimen made of ABS. However, compared to the fracture surface of the tensile specimen made of PLA, there are no visible layer boundaries. At the same time, Figure 15b shows no visible interlayer gaps or voids.

The resulting quality of layer-to-layer adhesion and mechanical properties of printed tensile specimens proves the high effectiveness of the proposed temperature control systemin comparison to the other optimization methods/techniques or tools listed in [2]. Results, obtained using such methods/techniques, are shown in [53,88,89,90,91,92,93,94,95,96,97] for specimens printed of ABS and [47,50,97,98,99,100,101,102,103,104,105] for the PLA ones.

Further research on 3D-printing with high-performance materials are planned, taking into account the results, recommendations and conclusions of other research studies, for example, [49,57,58,59,106,107].

Cooling rate characteristics of the induction heated (proposed) nozzle and the conventional hotend assembly (nozzle+heating block) at the extrusion speed of 0 mm/s are shown in Table 5.

Rapid heating can be achieved with aggressive PID tuning. At the same time, our previous article [63] represents the results of numerical modeling of physical processes or rather solution of thermal part of the multiphysics task. The nozzle temperature reached 300 °C at t = 4 s, f = 120 kHz with an initial temperature of 20 °C and fixed power consumption of 10%. The proposed manuscript presents several heating curves of the nozzle as an example. Figure 7a shows the nozzle temperature reached 750 °C on the fifteenth second of heating with a fixed power consumption of 80% and an operating frequency f = 120 kHz. Figure 7b shows the nozzle temperature reached 750 °C on the fifth second of heating with a fixed power consumption of 90% and operating frequency f = 120 kHz.

The calibration process for the proposed method was performed as a sequence of several experiments. We used a contact type thermometer UT325 with a bare K-type thermocouple as a reference measurement unit. Measurements were made in a steady-state in the operating temperature range from 50 to 350 °C. This temperature range meets the extrusion temperature requirements of most existing materials used in FDM 3D printing. 

The thermocouple wires were insulated with thin mica tubes (with a diameter small enough to allow the insertion of an insulated thermocouple into the 1.75 nozzle inlet) up to the junction point. In the first experiment, the thermocouple was placed inside the nozzle (in the polymer melt) at the relative heights of 90%, 50% and 10% from the nozzle bottom cut. The second experiment was done with the thermocouple soldered on the nozzle surface at the same relative heights. Results obtained during those experiments show a high amount of delay for the thermocouple measurements (both inside and outside of the nozzle) in comparison with the proposed indirect (eddy-current) method. In the case of the thermocouple being placed inside the nozzle, the observed time delay was about 5 s (showing the real speed of contact heating of the polymer melt with the given mass by the inner walls of the headed nozzle from 25 °C to the target temperature). Measured after stabilizing, this temperature was used as the reference one. In the case of the thermocouple being placed on the nozzle surface, the corresponding time delay was in the range from 0.6 to 1.2 s (a larger delay for the thermocouple being placed far from the center of the nozzle). It needs to be explicitly stated that the thermocouple is a local/point temperature measurement device, so wrong placing of the thermocouple (far from the most heated point of the nozzle surface, e.g., closer to the heatbreak or the nozzle outlet) results in the large overshoot during the heating (with the aggressive proportional–integral–derivative (PID) tuning, which allows high speed heating) or limits the heating speed. On the contrary, the proposed indirect (eddy-current) method measures the temperature of the exact same surface (exposed to the eddy currents) that is being heated. Thus, by design, this approach eliminates any additional delays introduced by the material properties (thermal conductivity of the nozzle material, etc.), wrong placement of the measurement sensors and so on. Additionally, the temperature (integral value over the whole nozzle surface) is measured for the same surface that is heated by the eddy currents (the surface over which eddy currents flow). This feature of the method eliminates the problems of thermal contact, as well as delays associated with the thermal conductivity of the heater material and sensor material (for contact measurement methods). Due to this feature of the method, the resulting estimate is integral. Details of the all experiments done with the target temperature of 250 °C are shown in Table 6.

According to the Multiphysics FEM model described in our previous article [63] an additional stage of modeling was completed with determination of a cooling rate of the preheated low weight nozzle at various extrusion speeds. The proposed nozzle was heated from 25 to 440 °C. After that, the nozzle was cooled for one second with such a variable parameter as extrusion speed. The corresponding parametric task was numerically solved using the ComsolMultiphysics modeling environment for the several values of extrusion speed (0, 40, 100, 200 mm/s).

**Figure 16:**
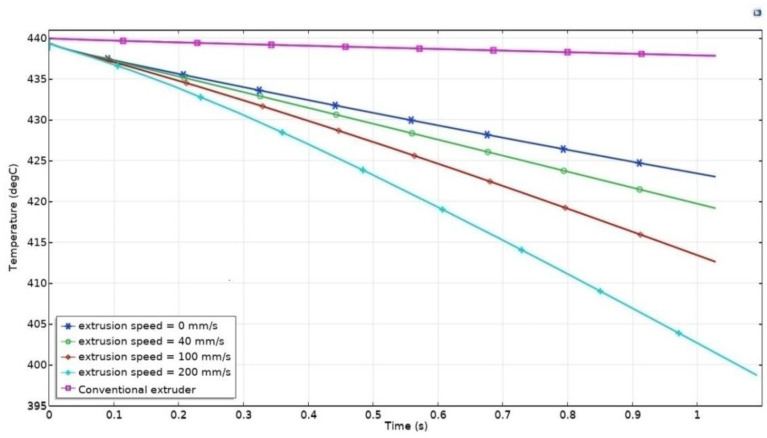
Cooling time curves of the induction heated (proposed)nozzle at various extrusion speeds and of the conventional hotend assembly (nozzle+heating block).

The results of numerical modeling of the physical processes at the initial nozzle temperature of 440 °C and extrusion speed of 0, 40, 100, 200 mm/s, t = 1 s, are shown in Figure 16. As can be seen, the cooling rate is the strong relation of extrusion speed.

In addition, the Multiphysics FEM model for the conventional extruder almost the same as the one described in [39] was formulated, and numerically solved using COMSOL 5.2a. The conventional hotend assembly (nozzle+heating block) was heated from 25 to 440 °C. After that, the nozzle was cooled for one second with such a variable parameter as extrusion speed. The corresponding parametric task was numerically solved using the ComsolMultiphysics modeling environment for the several values of extrusion speed (0, 40, 100, 200 mm/s). The results of numerical modeling of the physical processes at the initial conventional extruder temperature of 440 °C and extrusion speed of 0, 40, 100, 200 mm/s, t = 1 s, are shown in Figure 16. As can be seen, the cooling curves overlap each other, hence, there is extremely weak dependence of cooling rate on extrusion speed since the temperature sensor is installed in the heating block far away from the nozzle and extruding material, as already mentioned.

Based on obtained data, we could state that compared to conventional hotend assembly ramp rate of temperature versus time of induction heated nozzle is six times higher. This provides rapid cooling and heating of the proposed nozzle. In contrast with the conventional extruder, our induction heated nozzle allows one to control the heat input into the extruding material and already deposited layers of the printing object, to control viscosity of the extruding material and prevent spurious plastic efflux.

It is worth noting that the induction heated nozzle was cooled by 5 °C in 0.2 s: that is extremely fast. To ensure the required dynamic characteristics of heating and cooling processes in a high power induction heating system, an indirect (eddy-current) temperature measurement method is proposed.

As a summary, we could state that the proposed indirect (eddy-current) temperature measurement method has a number of important features:

1. There is no need for additional temperature sensors. The method is based on the analysis of the corresponding resonant circuit parameters;
2. It is a non-contact method of measuring temperature;
3. There is fast measurement due to a lack of inertial components and thermal contact conduction between the induction heating coil and the nozzle.

## 4. Conclusions

To ensure the required dynamic characteristics of heating and cooling processes in a high power induction heating system (peak power > 300 W), an indirect (eddy-current) temperature measurement method was proposed.

Fast and reliable temperature measurement of the low weight nozzle (<1 g) during high frequency induction heating was provided due to a lack of inertial components and thermal contact conduction between the induction heating coil (as well as sensing coil) and the nozzle.

An LCL series-parallel resonant circuit containing induction heating coil, sensing circuit, algorithms for digital signal processing (oversampling and exponential moving average filter), a regression model that determines the dependence of the desired signal on temperature and magnetic field strength were proposed to provide accurate measurement of the nozzle temperature.

The results of the completed experiment showed an absence of spurious plastic efflux and an absence of overheated areas with temperature-driven changes in filament color. With an accuracy of ±3 °C, the measurement time is 20 ms in the operating temperature range from 50 to 350 °C. The designed temperature control system based on an indirect measurement method will provide high mechanical properties and consistent quality of printed objects.

## Figures and Tables

**Table 1:** Values of “error” for nozzle temperature in the range from 25 to 750°C and fixed power consumption of 0.2, 10, 20, 100%.

Temperature °C	Error			
	0.2%	10%	20%	100%
110	90	83	75	36.5
120	96	88	80	42
250	201.3	182.3	164	74.5
280	224	202.5	184	84
400	320.2	290	262	118
440	353.7	320.3	289	129
750	601.3	542.5	489	215

**Table 2:** Fitting parameters for the regression model.

Coefficient	Estimate	Std Error
C	0.797	1.18
A	0.825	2.717 × 10^−3^
B	1.081 × 10^−4^	1.006 × 10^−4^
AB	−7.085 × 10^−6^	2.315 × 10^−7^
BB	4.951 × 10^−10^	1.982 × 10^−9^
ABB	−1.207 × 10^−10^	4.562 × 10^−12^

**Table 3:** Fused deposition modeling (FDM) 3D-printing process parameters.

Material	Nozzle Temperature °C	Bed Temperature °C	Raster Angle/No.		Extrusion Speed, mm/s	Nozzle Diameter, mm	Layer Thickness, mm
			−45°:45°	0°:90°			
Acrylonitrile butadiene styrene (ABS)	240	110	1	2	40	0.6	0.2
	250		3	4			
Polylactic acid (PLA)	210	60	1	2			
	220		3	4			

**Table 4:** The mechanical properties of tensile specimens printed of ABS and PLA.

Material	No.	Tensile Strength, MPa
ABS	1	43,58
	2	39,44
	3	36,71
	4	37,16
PLA	1	59,61
	2	64,47
	3	62,64
	4	60,91

**Table 5:** Cooling rate characteristics of the induction heated (proposed) nozzle and the conventional hotend assembly (nozzle+heating block) at the extrusion speed of 0 mm/s.

Initial Temperature/Final Temperature °C	Induction Heated (Proposed) Nozzle, Cooling Time, s	Conventional Hotend Assembly (Nozzle + Heating Block), Cooling Time, s
300/250	5	34
250/230	3	22
250/200	9	60
200/150	13	80
150/100	22	120
100/50	41	250
250/50	82	450

**Table 6:** Temperature measurement (experimental) results.

	Target Temperature, °C	Steady State (After 30 s, Averaged over 5 s), °C	Value Fluctuations (10 Consecutive Measurements after 30 s, in 5 s. Intervals, No Averaging), °C	Setting Time after Disabling Heater, s	Max Overshoot, °C
Proposed method	250	248.1	247.9–248.2	0.3	1.3
Thermocouple inside, 10%		236.3	236.2–236.3	12.8	26
Thermocouple inside, 50% (reference)		250.1	250.0–250.2	6.3	11
Thermocouple inside, 90%		221.4	221.3–221.6	18.5	38
Thermocouple on surface, 10%		235.4	235.3–235.5	3.2	6
Thermocouple on surface, 50%		250.0	249.9–250.1	2.5	4
Thermocouple on surface, 90%		216.5	216.4–216.7	4.8	8

## Data Availability

The data presented in this study are available on request from the corresponding author. The data are not publicly available due to the fact that further study will be carried out using the same data.
